# Optimizing CRISPR/Cas9 Editing of Repetitive Single Nucleotide Variants

**DOI:** 10.3389/fgeed.2022.932434

**Published:** 2022-07-05

**Authors:** Inga Usher, Lorena Ligammari, Sara Ahrabi, Emily Hepburn, Calum Connolly, Gareth L. Bond, Adrienne M. Flanagan, Lucia Cottone

**Affiliations:** ^1^ Department of Pathology (Research), UCL Cancer Institute, University College London, London, United Kingdom; ^2^ Department of Haematology, UCL Cancer Institute, University College London, London, United Kingdom; ^3^ UCL Medical School, University College London, London, United Kingdom; ^4^ Institute of Cancer and Genomic Sciences, University of Birmingham, Birmingham, United Kingdom; ^5^ Department of Histopathology, Royal National Orthopaedic Hospital, London, United Kingdom

**Keywords:** CRISPR, CRISPR/Cas9, genome editing, prime editing, homology directed repair (HDR), cell line, stem cells

## Abstract

CRISPR/Cas9, base editors and prime editors comprise the contemporary genome editing toolbox. Many studies have optimized the use of CRISPR/Cas9, as the original CRISPR genome editing system, in substituting single nucleotides by homology directed repair (HDR), although this remains challenging. Studies describing modifications that improve editing efficiency fall short of isolating clonal cell lines or have not been validated for challenging loci or cell models. We present data from 95 transfections using a colony forming and an immortalized cell line comparing the effect on editing efficiency of donor template modifications, concentration of components, HDR enhancing agents and cold shock. We found that *in silico* predictions of guide RNA efficiency correlated poorly withactivity in cells. Using NGS and ddPCR we detected editing efficiencies of 5–12% in the transfected populations which fell to 1% on clonal cell line isolation. Our data demonstrate the variability of CRISPR efficiency by cell model, target locus and other factors. Successful genome editing requires a comparison of systems and modifications to develop the optimal protocol for the cell model and locus. We describe the steps in this process in a flowchart for those embarking on genome editing using any system and incorporate validated HDR-boosting modifications for those using CRISPR/Cas9.

## 1 Introduction

CRISPR-associated protein 9 (Cas9) genome editing systems have revolutionized biological research. The ability to target and modify genetic loci offers the potential to investigate the functional consequences of variants discovered through genome wide association studies (Gallagher and Chen-Plotkin, 2018; Chen et al., 2022) and to correct the >75,000 known human pathogenic variants listed in Clinvar (Landrum and Kattman, 2018). The original CRISPR/Cas9 system has been available for more than a decade and extensive research has characterized how CRISPR/Cas9 works and how this can be optimized for maximal efficiency, with over 18,000 publications related to the term “CRISPR” listed on PubMed. CRISPR/Cas9 components are widely commercially available as recombinant components and plasmids. It is possible to achieve efficiencies of up to 80% in human cells when utilizing CRISPR/Cas9 to disrupt the DNA sequence and generate gene knock-outs (Guo et al., 2018). However, when using CRISPR/Cas9 to substitute nucleotides (knock-in), the efficiency is far lower, often in the order of 1%, although efficiencies of up to ∼50% are reported following protocol modifications (Paquet et al., 2016; Kwart et al., 2017; Guo et al., 2018; Okamoto et al., 2019; Maurissen and Woltjen, 2020) (reviewed in Liu et al., 2019a). More recently, it was demonstrated in yeast that retron-based CRISPR/Cas9 could boost multiplexing knock-in experiments, opening new possibilities for human cells (Zhao et al., 2022). Nevertheless nucleotide substitution by CRISPR/Cas9 relies on the non-dominant DNA repair pathway, homology directed repair (HDR). CRISPR/Cas9’s reliance on DNA damage repair limits the efficiency of substituting nucleotides and can introduce off-target mutations.

Prime editing (PE) emerged in 2019 to address the low knock-in efficiency and propensity for off-target effects of CRISPR/Cas9 and is available in its latest iteration with PE4 and 5 (Anzalone et al., 2019; Chen et al., 2021). PE utilizes the guide RNA-directed “search” ability of CRISPR/Cas9 but builds on the accuracy of the “replace” ability by avoiding a double stranded break and reliance on cellular DNA repair. Base editors represent another alternative to CRISPR/Cas9 for single nucleotide substitution but are limited by their editing of bystander nucleotides making them unsuitable for repetitive nucleotides (Rees and Liu, 2018). Together, CRISPR/Cas9, PE and base editors comprise the main genome editing toolbox available to the contemporary researcher.

The efficiency of genome editing varies with the target locus, distance to the protospacer adjacent motif (PAM) and mismatch repair proficiency of the cell model (Chen et al., 2021; Ferreira da Silva et al., 2022). For example, the editing efficiency of CRISPR/Cas9 falls as distance from the PAM increases and stem cells show relative resistance to transfection compared to immortalized cell lines (Madsen and Semple, 2019). PE shows improved efficiency and reduced off-target changes compared to CRISPR/Cas9, however PE necessitates delivery as a plasmid, the design of more components (pegRNA spacer, extension and ngRNA for PE3b) and more extensive optimization compared to CRISPR/Cas9. PE efficiency is highly variable (up to 50-fold difference) depending on the genetic background of the cell model and component design (Anzalone et al., 2019). These factors make it likely that CRISPR/Cas9-mediated HDR will still be employed for repetitive target loci or cell models that are not amenable to PE, where a plasmid delivery is not desirable or where the rate of CRISPR/Cas9-mediated HDR is sufficiently high to avoid the need for extensive design and optimization processes. Selecting the right system for an experiment will depend on the target locus, intended substitution and cell model.

In this study we edited three single nucleotide loci implicated in the development of sarcomas using the CRISPR/Cas9 system. These loci are challenging to edit because they are repetitive, precluding the use of base editors, and distant from a PAM (>15 nucleotides), making editing by CRISPR/Cas9 or other Cas enzymes challenging. We applied several previously reported optimizing modifications across 95 transfections in induced pluripotent stem cells (iPSC), representing a colony forming model, and an adherent cell model to validate their utility in editing these challenging loci. Editing outcomes were characterized at each stage using contemporary technologies. While PE was not available at the time of our experiments, we have addressed how we would incorporate its use. Finally, we synthesize a flowchart that can be adapted to any subsequent CRISPR genome editing system.

## 2 Materials and Methods

### 2.1 Induced Pluripotent Stem Cells

#### 2.1.1 Cell Culture

The human episomal line of induced pluripotent stem cells (A18945, Gibco; Thermo Fisher Scientific, Inc, Waltham, MA, United States ) was maintained in feeder-free culture on Geltrex (A1413202, Invitrogen, Thermo Fisher Scientific) and Essential 8 Flex (E8 Flex) medium (A28585, Gibco, Thermo Fisher Scientific) supplemented with 0.5% of Penicillin (10,000 U/ml). Cells were cultured at 37 °C in a humidified atmosphere with 5% CO2. Cells were passaged by incubation for 5 min at 37 °C with Dulbecco’s phosphate buffered saline (DPBS)-EDTA 0.5 mM pH 8.00 (Invitrogen, Thermo Fisher Scientific, 14190250 and 15575020).

#### 2.1.2 CRISPR/Cas9 Editing of iPSC

All CRISPR/Cas9 components were purchased through IDT. Single-stranded oligodeoxynucleotides (ssODN) were ordered as Alt-R™ HDR Donor Oligos.

1. The gRNA was prepared by duplexing Alt-R® CRISPR-Cas9 crRNA and Alt-R® CRISPR-Cas9 tracrRNA (IDT, 1072532).
2. The ribonucleoprotein (RNP) was formed using 0.78 μl Alt-R® CRISPR-Cas9 gRNA, 1.02 μl Alt-R® S. p. Cas9 Nuclease V3 (IDT, 1081058) and 1.2 μl PBS (total 3 μl).
3. Cells were detached, counted and transfected using the Lonza™ P3 Primary Cell 4D-Nucleofector™ X (Lonza, V4XP-3024) and electroporation program CA137.
4. To prepare the electroporation mixture, 0.5 × 10^6^ cells were resuspended in 20 μl Lonza electroporation buffer, 1 μl of RNP complex and 0.5 μl Alt-R™ HDR Donor Oligo.
5. Following transfection, cells were recovered: (i) at 37°C, (ii) at 32 °C for 24 h then at 37 °C, (iii) with Alt-R® CRISPR-Cas9 HDR enhancer (IDT, 1081072) in E8 Flex without antibiotics for 24 h, (iv) with DMSO at 1% in E8 Flex without antibiotics for 24 h.

#### 2.1.3 Colony Picking

For all stressful steps, such as single cell dissociation and colony picking, E8 Flex medium with RevitaCell™ was used for 2 h before and until colonies formed. In all other steps E8 Flex was used without RevitaCell™.

Cells were detached with Accutase® (Innovative Cell Technologies, Inc, San Diego, CA AT104), counted and 700–1,000 cells per dish were plated. Medium-sized colonies were picked using a P200 pipette. The aspirated colony was transferred to a 96 well plate and triturated 10 times. When cells were 50–70% confluent they were split 1:2, half were seeded for genomic DNA extraction and half for subculture or freezing.

#### 2.1.4 Mirror Plates for Freezing and for DNA Extraction

Colonies expanded in 96 well plates were washed with 100 μl DPBS and detached with 30 μl of Accutase®. A mirror plate for freezing was prepared containing 50 μl of 2X freezing medium (E8 Flex plus 20% DMSO). Cells were collected with 70 μl of E8 Flex and 50 μl were transferred to the mirror plate and stored at −80°C. The remaining 50 μl of suspension was kept for DNA extraction: the plate was spun at 1950 RCF for 30 min at 4 °C, the medium was removed, and the plate stored at −80°C. Upon thawing, 30–50 μl of Lucigen QuickExtract™ DNA Extraction Solution (LGC, Middlesex, United Kingdom, QE09050) was added, then triturated and heated at 65°C for 6 min then 98°C for 2 min and used directly for genotyping.

### 2.2 U-CH1 Chordoma Cell Line

#### 2.2.1 Cell Culture

The human U-CH1 chordoma cell line (ATCC® CRL-3217™, www.chordomafoundation.org) was grown as previously described (Scheipl et al., 2016). Cell authentication was regularly performed by Short Tandem Repeat fingerprinting (Culture Collections, Public Health England, United Kingdom) (Supplementary Table 2).

#### 2.2.2 CRISPR/Cas9 Editing

1. The gRNA was prepared by duplexing Alt-R® CRISPR-Cas9 crRNA and Alt-R® CRISPR-Cas9 tracrRNA (IDT, 1072532).
2. 3.9 μl Alt-R® CRISPR-Cas9 gRNA, 5.1 μl of 10 mg/ml (or 10 μg/μl = 50 μg = 300 pmol) Alt-R® S. p. Cas9 Nuclease V3 (IDT, 1081058) and 5.9 μl sterile DPBS (total 15 μl) were combined for the RNP.
3. Cells were detached, counted, and transfected using the Lonza Amaxa® Cell Line Nucleofector® Kit V (Lonza, Basel, Switzerland VCA-1003) using electroporation program A30. To prepare the electroporation mixture, 2 × 10^6^ cells were resuspended in 60 μl Lonza electroporation buffer, with 10 μl of RNP complex (final Cas9 concentration ∼100 pmol) and 3 μl of modified Alt-R™ HDR Donor Oligo.
4. Following transfection, the cells were recovered in medium without antibiotics.

#### 2.2.3 Analysis of editing outcomes using digital droplet polymerase chain reaction (ddPCR)

A common primer set and probes for each allele were designed: a hexachlorofluorescein (HEX) probe for the parental allele (A/T) and fluorescein amidite (FAM) probe for the edited allele (G/C). ddPCR assays were designed using primer3plus (Untergasser et al., 2007) and the BioRad Droplet Digital™ PCR Applications Guide. ddPCR experiments were carried out using the BioRad QX200 ddPCR supermix for probes (no dUTP) workflow, Automated Droplet Generator, BioRad Automated Droplet Generation Oil for Probes (BioRad, Hercules, California, United States; #1864110), Eppendorf vapo. protect thermocycler and QX200 Automated Droplet Reader. Results were analyzed using the BioRad QuantaSoft™ Analysis Pro Software using rare event detection.

#### 2.2.4 Flow Cytometry Activated Cell Sorting (FACS)

Transfected U-CH1 cells were recovered for 36 h before single cell sorting into collagen-coated 96 well plates using a BD FACS Aria Fusion Cell Sorter™ (Becton Dickinson, Franklin Lakes, New Jersey, United States ) running FACSDiva Software version 6. Cells were incubated with TOPRO3+ (Garvey et al., 2016) before sorting to allow the exclusion of dead cells and the top 10% of ATTO-550 positive cells were selected.

#### 2.2.5 Mirror Plate for DNA Extraction

Cells were washed with 100 μl DPBS and detached with 50 μl of Accutase® (Innovative Cell Technologies, AT104) at 37 °C for 10–15 min 50μl of medium was added, 50 μl were taken for genomic DNA extraction using Lucigen QuickExtract™ (LGC, QE09050) while the other 50 μl were replated for subculture.

### 2.3 Techniques Common to Both Cell Models

Regular testing was performed to exclude *mycoplasma* contamination using the EZ-PCR *Mycoplasma* Test Kit (K1-0210, Geneflow, Lichfield, Staffordshire, United Kingdom).

#### 2.3.1 Genotyping Using Illumina MiSeq™ Next Generation Sequencing (NGS)

DNA was extracted using Zymo Column Extraction (Zymo Research, Irvine, California, United States , D3024) (for bulk transfections) or Lucigen QuickExtract™ DNA Extraction Solution (for picked colonies). PCR was performed with Kapa Hifi HotStart polymerase (for <500 base pair product) (Kapa Biosystems, Roche Molecular Systems, Inc, Pleasanton, California, United States , KR0370): 12.5 μl 2X KAPA HiFi HotStart ReadyMix, 0.75 μl 10 μM Forward Primer (with MiSeq™ adapter, Supplementary Table S3), 0.75 μl 10 μM Reverse Primer (with MiSeq™ adapter), 2 μl DNA and PCR-grade water up to 25 μl. 3 μl of DNA extracted in Lucigen was used for PCR. The PCR products were purified using the QIAquick PCR Purification Kit (QIAGEN Ltd, Manchester, England). MiSeq™ was performed in-house.

#### 2.3.2 Analysis of Editing Outcomes Using MiSeq™ Data

FASTQ files were analyzed using Cas Analyser (Park et al., 2017) with the following parameters: Nuclease type = single nuclease, comparison range (R) = 40, Minimum frequency (*n*) = 1 and no optional wild type marker. Rates of unedited and edited outcomes (NHEJ ± substitution and HDR) were calculated by number of reads containing outcome/total number of reads. Indels were changes in sequence length compared to the reference sequence.

We defined the outcomes as follows:

• Wild type/unedited (90–100% of reads match the reference sequence)
• (Homozygous) knock-out (90–100% of reads show indels)
• Heterozygous knock-in (40–60% of the reads match the reference and 40–60% show the knock-in)
• Homozygous knock-in (90–100% of reads show the knock-in)
• Combined/mixed repair (40–60% of the reads show indels and 40–60% match the reference sequence or show the knock-in).

#### 2.3.3 Genotyping Using Sanger Sequencing

PCR was performed: 12.5 μl AmpliTaq Gold™ 360 Master Mix (Thermo Fisher Scientific, 4398881), 0.5 μl 10 μM forward-reverse primer mix (Supplementary Table S3), 10 μl water plus 2 μl Lucigen QuickExtract™ DNA. PCR products were cleaned using the ExoSAP-IT™ Express PCR Product Cleanup Reagent (Applied Biosystems, Thermo Fisher Scientific, 15563677) and sent for Sanger sequencing (Source BioScience, Nottingham, United Kingdom).

#### 2.3.4 Genotyping Using TaqMan™ qPCR

TaqMan™ genotyping was performed for rs2305089 (Applied Biosystems, 4351379): 5 μl TaqMan™ genotyping mastermix, 0.5 μl Taqman™ primer/probe mix (C__11223433_10), 3.5 μl water, 1 μl DNA. Results were analysed using the Genotyping application on the Thermo Fisher Connect™ cloud.

### 2.4 Data Analysis

All analysis and statistics were performed using R version 4.0.5 (2021–03–31) and GraphPad Prism version 8.0.0 for Windows (GraphPad Software, San Diego, California United States , www.graphpad.com). Cartoons were created with Biorender.com.

## 3 Results

### 3.1 Editing of *TP53* SNVs in iPSC or Other Colony Forming Cell Model

**FIGURE 1:**
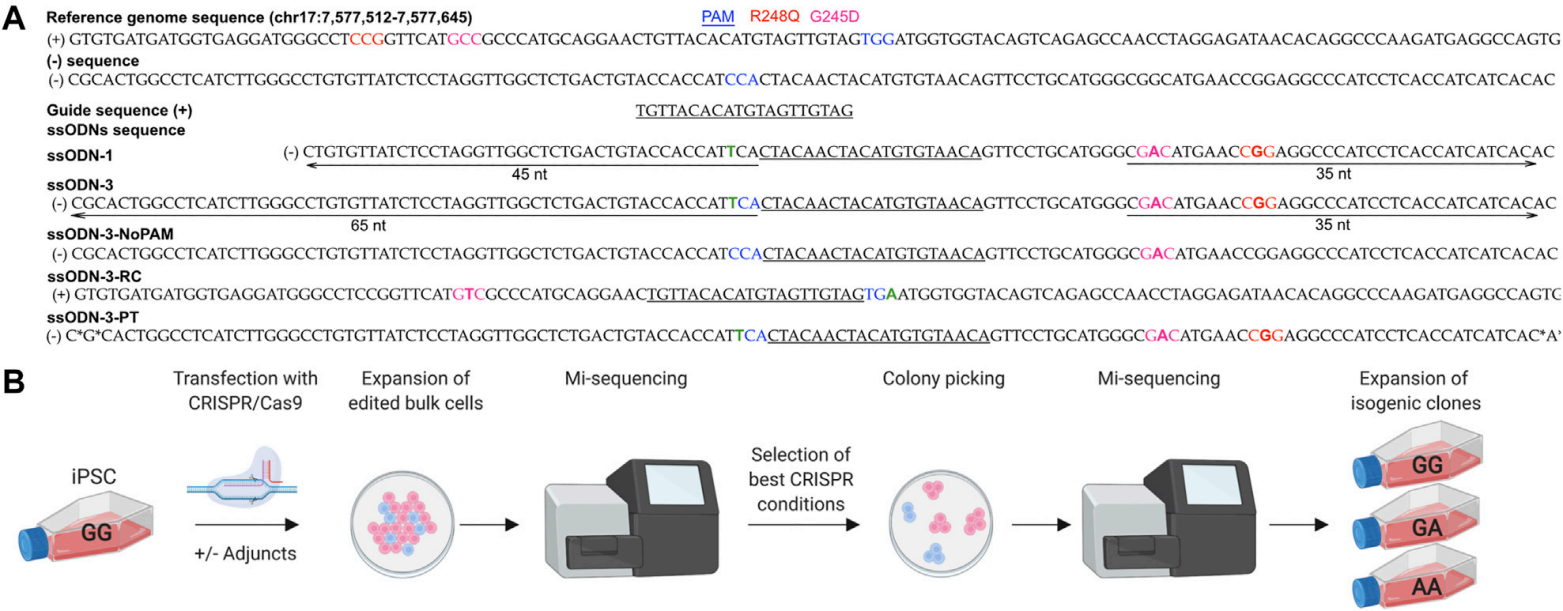
Workflow for early characterization of bulk populations by MiSeq followed by clonal line isolation by colony picking. **(A)** Design of CRISPR/Cas9 components. Five ssODN designs were compared. *: phosphorothioate bonds. Green “T” indicates PAM modification to prevent cleavage of the donor. **(B)** iPSCs were transfected under different experimental conditions and screened by MiSeq NGS. Populations with the highest rates of HDR were selected for colony picking. Individual colonies showing accurate repair by MiSeq were expanded as isogenic lines.

We tested the ability of CRISPR/Cas9 modifications to introduce the germline pathogenic G245D and R248Q variants in *TP53* which lie in proximity, distant from a PAM and in repetitive sequences, into iPSC (Figure 1A). iPSCs tolerate transfection and single cell sorting poorly and are expensive to maintain in culture making it important to characterize editing outcomes early in the workflow (Figure 1B). *TP53* is expressed in iPSCs making it likely that the chromatin will be open allowing access of the RNP (Liu et al., 2019b).

### 3.2 Preliminary Checks of Cell Model

iPSCs accumulate genetic alterations during cell culture including SNVs at G245D, R248Q and other loci in *TP53* (Amps et al., 2011; Merkle et al., 2017; Liu et al., 2019b). We therefore first ensured that a genetically pure population, free of *TP53* SNVs, was utilized for transfection by picking 20 clonal sublines grown from the parental iPSC line; and checked five by Sanger sequencing which were found to be free of SNVs in the region around the G245D and R248Q loci. One of these sublines was selected for transfection (Supplementary Figure S1).

### 3.3 Design of Components Targeting *TP53* in iPSCs

We designed 6 guide RNAs (gRNAs) for CRISPR/Cas9 using online tools E-CRISP Heigwer et al., 2014, CHOPCHOP Labun et al., 2019 and the IDT Alt-R™ CRISPR HDR Design Tool (https://eu.idtdna.com/pages/tools/alt-r-crispr-hdr-design-tool) (Supplementary Figure S2B). All gRNAs were comparable in their likelihood of on- and off-target effects when assessed *in silico.* When tested in cells, only one gRNA generated a double strand break (DSB) on Sanger sequencing (Supplementary Figure S2C and Figure 1A) and did so at >15 nucleotides from the SNVs limiting our choice of gRNA and editing efficiency (Paquet et al., 2016; Kwart et al., 2017) (Figure 1A and Supplementary Figure S2B).

Designing PE components is more complex. PegRNA design is a major factor determining editing efficiency and several primer binding sites (PBS) and reverse transcriptase (RT) template combinations are possible when designing the pegRNA but only a fraction of these will achieve optimal efficiency (Anzalone et al., 2019). Various tools have been developed to aid design including PEGfinder (Chow et al., 2020), PE-designer (Hwang et al., 2021) and PrimeDesign (Hsu et al., 2021). For the G245D locus in *TP53*, 19 pegRNA designs were generated using PrimeDesign which could be combined with >10 PE gRNAs (Hsu et al., 2021) (data not shown). However, the low tolerance of iPSC to multiple transfections discouraged the use of the PE system for this study.

### 3.4 Optimizing the HDR Efficiency of CRISPR/Cas9 in iPSCs

HDR-enhancing modifications to the CRISPR/Cas9 protocol have been extensively investigated (Supplementary Table S1). As we could not modify the cut-to-mutation distance, we tested if modifying ssODN design or post-transfection experimental conditions would improve the rate of HDR. We performed 75 individual transfections of iPSCs (Figures 1A,B) (Supplementary Table S1) which tolerated electroporation with 40–50% viability after transfection, and compared editing outcomes in the bulk populations using MiSeq (reviewed in Sledzinski et al., 2020).

**FIGURE 2:**
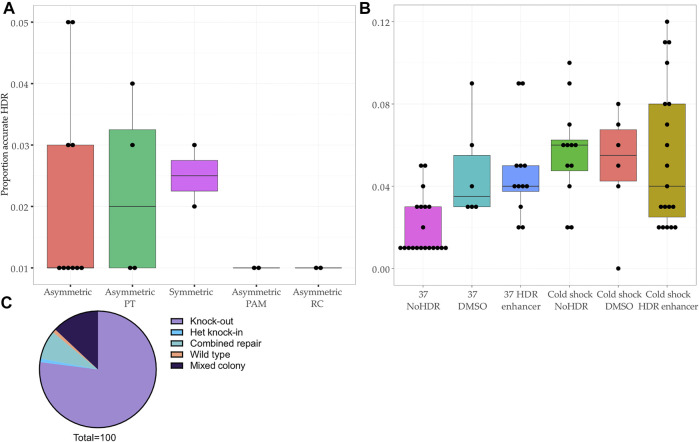
Comparison of HDR efficiency associated with protocol modifications at the bulk population level and editing outcomes in clonal lines. **(A–B)** Proportion of reads showing accurate repair by HDR in bulk population DNA transfected with **(A)** different ssODNs (*p*-value <0.45, Kruskal–Wallis test) and **(B)** different experimental conditions (*p*-value < 0.01, Kruskal–Wallis test). Asymmetric PAM: asymmetric donor without blocking mutation in PAM. Asymmetric PT: addition of phosphorothioate nucleotides to asymmetric ssODN. Asymmetric RC: reverse complement of asymmetric ssODN. HDR: addition of Alt-R™ HDR Enhancer after transfection. DMSO: addition of DMSO after transfection. NoHDR: no HDR enhancer or DMSO after transfection. **(C)** Editing outcomes in 100 colonies picked from two transfections showing population HDR rates of 11 and 12%.

The editing efficiency was variable, ranging from 0 to 12% for the knock-in without indels (accurate HDR) (Figures 2A,B). We corroborated previous reports (Richardson et al., 2016; Liang et al., 2017; O’Brien et al., 2019) that asymmetry of the homology arms improves accurate HDR (range 1–5%, mean 2.0%, 10 samples) as did addition of phosphorothioated nucleotides (range 1–4%, mean 2.3%, 4 samples) (Papaioannou et al., 2009; Gutierrez-Triana et al., 2018) and arms of equal length (range 2–3%, mean 2.5%, 2 samples) (Figure 2A). The introduction of a silent mutation of the PAM (Paquet et al., 2016; Okamoto et al., 2019), or the reverse complement (RC) of the asymmetric design (Richardson et al., 2016) (Figure 1A, Supplementary Table S1 and Supplementary Table S3) did not improve the HDR efficiency (Figure 2A), however only two experiments were performed for these conditions.

Next, using the asymmetric ssODN, we proceeded to modify post-transfection conditions by adding 1% dimethyl sulfoxide (DMSO) or the IDT Alt-R™ HDR Enhancer (Stratigopoulos et al., 2018) and culturing cells at 32°C (cold shock) (Guo et al., 2018) (Supplementary Table S1). The most effective protocol included cold shock and Alt-R™ HDR Enhancer, a finding consistent with previous studies (Skarnes et al., 2019; Di Stazio et al., 2021) (Figure 2B). We confirmed that DMSO increases HDR, making it a cost-effective alternative to commercial HDR enhancers (Stratigopoulos et al., 2018) (Figure 2B).

### 3.5 Isolating Cell Lines Reduces the Editing Efficiency Observed in the Bulk Population by NGS

We chose two transfected populations which showed a promising rate of accurate HDR (11 and 12%) for single cell line isolation (Figure 1B and Figure 2C). After colony picking and expansion, most colonies were repaired by NHEJ (77/100, 77%) or showed a mixed repair (13/100, 13%). The final editing efficiency was 1% despite the initially promising rate of HDR at the level of the bulk population.

Finally, quality assurance of the clonal lines was undertaken to assess the homogeneity/purity of the population. Using MiSeq we established that the clonal lines were pure populations free of off-target alterations in the ∼200 base pairs surrounding the variants. No off-target sites were predicted by the gRNA design tools.

Our data show that *in silico* predictions of gRNA efficiency correlated poorly with activity in our iPSC. Rates of HDR vary between transfections and can be boosted with asymmetric donors, with or without PT modifications, and HDR-enhancing modifications. The isolation of clonal cell lines from bulk populations resulted in a significant attrition of HDR efficiency.

### 3.6 Editing of *TBXT* in the U-CH1 Immortalized Cancer Cell Line

Many cell models grow as adherent cultures that tolerate single cell sorting and are relatively cheap to culture. The emphasis for these models is high throughput generation and genotyping of cell lines. U-CH1 is a cell model of the rare bone cancer, chordoma, and is associated with the G177D SNV in *TBXT*. We employed the U-CH1 chordoma cell line, which expresses *TBXT* at high levels and likely to be in euchromatin, to investigate the functional impact of the G177D SNV (Kelley et al., 2010; Pillay et al., 2012).

### 3.7 Design of CRISPR/Cas9 Components for the U-CH1 Chordoma Cell Line

We ensured a pure population free of SNVs in the region surrounding the G177D variant using MiSeq to avoid the time taken for single cell sorting (Supplementary Figure S4A).

**FIGURE 3:**
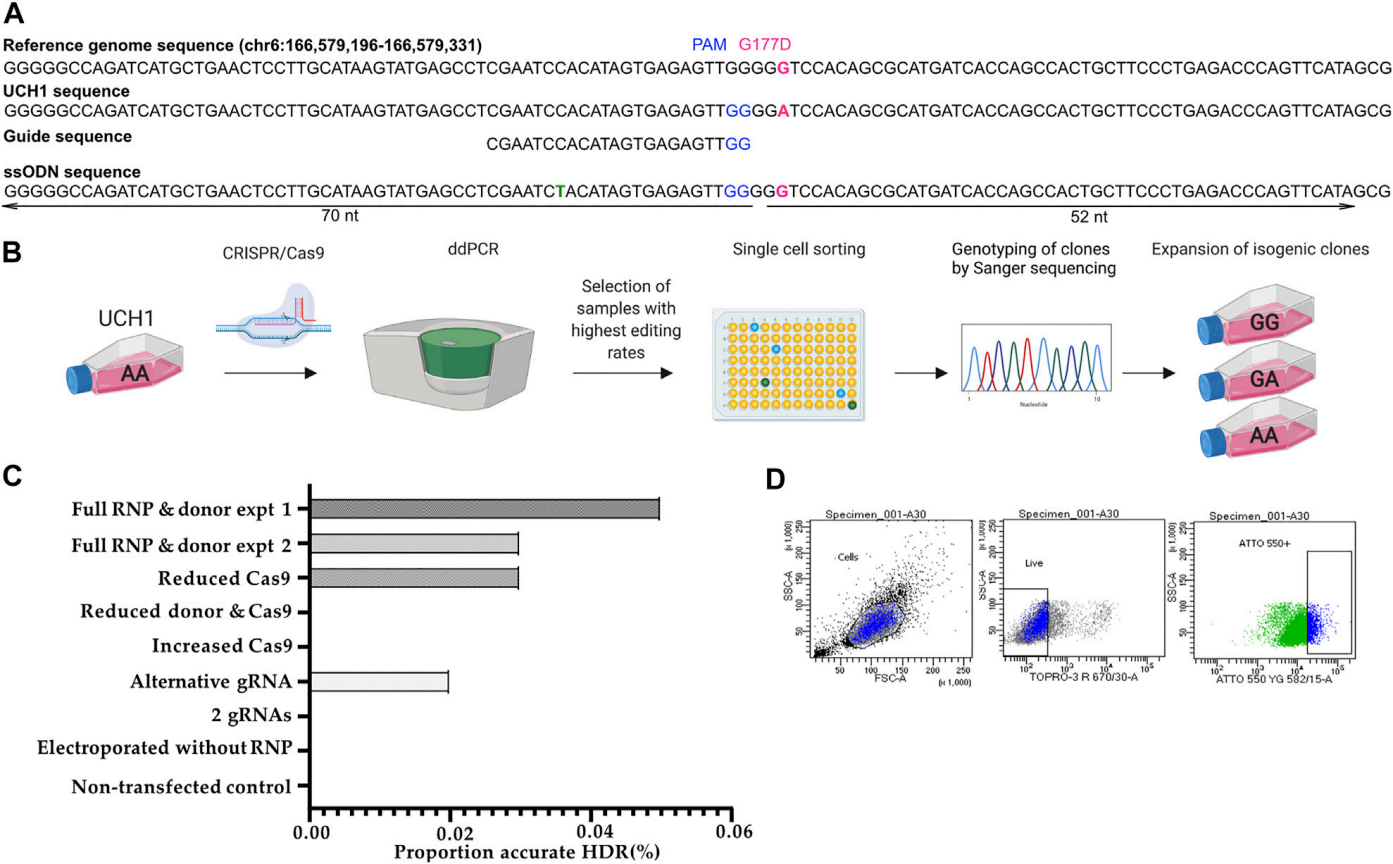
Workflow for early single cell sorting and high throughput genotyping. **(A)** Design of CRISPR/Cas9 components targeting the G177D SNV in *TBXT*. **(B)** After transfection, U-CH1 bulk populations were screened by ddPCR. Populations showing HDR (2–10%) were single cell sorted using FACS. DNA was extracted from expanded clonal cell populations by establishing a “mirror plate” and used directly for genotyping by TaqMan™ qPCR or ddPCR **(C)** Bar plot of proportion of reads showing accurate HDR when concentrations of CRISPR/Cas9 components are varied. **(D)** Dot plots showing the gating strategy for sorting U-CH1 cells based on TOPRO3 and ATTO-550 fluorescence.

Four candidate gRNAs were designed and assessed using the IDT CRISPR-Cas9 guide RNA design checker. Of the four gRNAs predicted to be effective *in silico*, two caused a DSB when tested in cells (Figures 3A,B and Supplementary Figure S4B–C).

We tested whether varying Cas9 (2.05 μl (17 pmol final concentration) versus 10.2 μl (404 pmol final concentration)), donor template concentration or combining gRNAs would improve the editing efficiency as measured by MiSeq (Lin et al., 2014). We established that the RNP complexed with a single gRNA, at recommended concentrations, showed the best performance (Figure 3C).

### 3.8 Screening of Transfected Bulk Populations Using Digital Droplet PCR

After transfection we screened the bulk population for HDR using digital droplet PCR (ddPCR) as a faster alternative to MiSeq before proceeding to single cell sorting, allowing us to maintain the cells in culture during screening as U-CH1 cells grow slowly (Figure 3B). The partitioning technology of ddPCR enables the detection of rare knock-in events at a frequency as low as 0.1% (Miyaoka et al., 2014, Miyaoka et al., 2018). Screening of 10 transfected populations by ddPCR showed an editing efficiency of 2–10%. The transfected cells tolerated single cell sorting by FACS 24–36 h after transfection (Figure 3B) then required 3–5 weeks to expand sufficiently for subculture into a “mirror” plate for genotyping. The Lucigen one-step DNA extraction protocol was utilized, allowing direct input of DNA into TaqMan™ qPCR or ddPCR for high throughput genotyping.

### 3.9 High Throughput Screening of Hundreds of U-CH1 Clones

We screened ∼500 transfected clonal lines and isolated seven cell lines which were free of indels: four wild type, two heterozygous knock-ins and one homozygous knock-in, giving an overall HDR efficiency of 0.6% (Figure 3D).

The relationship between HDR efficiency and cut-to-mutation distance (Paquet et al., 2016; Kwart et al., 2017) was again observed: the silent blocking mutation introduced into the ssODN was successfully edited more frequently than the G177D SNV and in a homozygous fashion, in contrast to the G177D SNV which was only edited in one allele.

Finally, we checked the isolated clones by sequencing 1,000 bp around the site of the edit by Sanger sequencing: we confirmed the presence of the introduced edit and ensured no off-target alterations were present (Supplementary Figure S6). No other off target sites were predicted by the design tools.

In summary, we show the results of screening hundreds of potential edited clones using high throughput technologies but highlight a significant attrition in efficiency between bulk populations and single cell line isolation. Varying the concentration of CRISPR/Cas9 components did not improve editing efficiency in U-CH1.

## 4 Discussion

**FIGURE 4:**
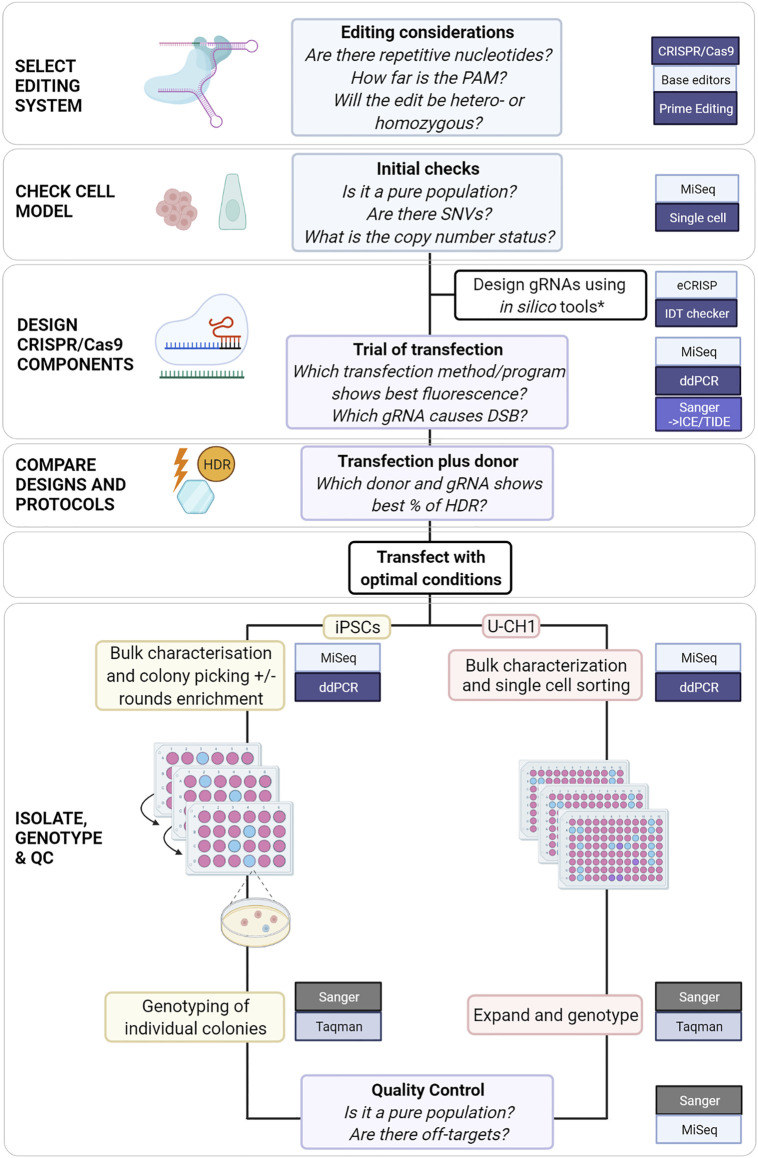
Proposed comprehensive flowchart for editing stem cells or immortalized cell lines using CRISPR/Cas9. *(Heigwer et al., 2014; Labun et al., 2019; Sledzinski, Nowaczyk and Olejniczak, 2020) and (Schubert et al., 2021) for HDR-specific ssODN design.

When planning a genome editing experiment, the methods available to the scientist have expanded substantially over the past decade. Each system, CRISPR/Cas9, base editors and PE, offers solutions for introducing different alterations: CRISPR/Cas9 for knock-outs and knock-ins, base editors for substitutions in non-repetitive nucleotides and PE for substitutions, transversions and indels. As genome editing systems require increasingly complex components and modified systems emerge, preliminary experiments become increasingly important for informing final experimental design (Anzalone et al., 2019). We propose a flowchart that incorporates critical steps and tools utilized at each stage to edit colony-forming cells or adherent cell lines (Figure 4).

Bioinformatic platforms are indispensable for designing CRISPR/Cas9 components but the accuracy of predictions can be affected by the chromatin organization of the target locus and varies by cell model. This was confirmed by our results that show that *in silico* predictions of gRNAs efficiency did not show the same performance in cells. Even when experiments are repeated there was several-fold variation in the HDR efficiency, potentially related to factors such as inherited genetic variability and cell cycle phase, highlighting the importance of characterizing bulk populations before single cell line isolation.

Cut-to-mutation distance is a major factor in determining CRISPR/Cas9 HDR efficiency and zygosity of the resulting edit (Paquet et al., 2016; Kwart et al., 2017). This was confirmed in our experiments with the frequent homozygous knock-in of the silent PAM mutation, close to the cut site, compared to the heterozygous knock-in of the target locus, distant from the cut site. This may have accounted for the successful homozygous knock-in in U-CH1 where the mutation is closer to the cut site than in iPSCs. If a homozygous knock-in is required, gRNAs that cut close to the target locus are required. If these gRNAs are not effective in preliminary testing, it is worthwhile considering Cas9 nucleases with different PAM requirements or using PE. We were able to validate the beneficial effect of an asymmetric donor, HDR enhancer and cold shock, the combination of which increased the low baseline HDR efficiency several fold. Asymmetric donors are thought to influence annealing and release of strands being repaired (O'Brien et al., 2019) while HDR enhancers block NHEJ (Pinder, Maurissen, reviewed in Bischoff) to favor HDR and cold shock is thought to affect G2/M transition or persistence of the RNP (Guo, Maurissen). Tools that aid optimal design of ssODNs may be employed (O’Brien et al., 2019; Schubert et al., 2021).

Despite these optimizations boosting the HDR efficiency to a promising mean rate of 11.5% in the bulk population, a single cell line was isolated from transfected iPSCs with a similar picture in U-CH1. This may be related to the low overall editing efficiency, consistent over repeated transfections, compared to previous optimization studies. Although some studies showed impressive rates of HDR, it is important to note that they instead utilized a bulk population-based read out without isolating cell lines (Zhang et al., 2017; Skarnes et al., 2019; Di Stazio et al., 2021) or they edited the more robust HEK293 cell model (Richardson et al., 2016; Liang et al., 2017; Di Stazio et al., 2021). The significant attrition of editing efficiency is an important consideration when modest editing efficiencies are achieved.

Characterization of the factors affecting the efficiency of PE is less advanced but initial studies suggest optimizing the melting temperature of the PBS, using a dual pegRNA strategy (Lin et al., 2021) and disrupting the action of the mismatch repair pathway (Ferreira da Silva et al., 2022). Analysis of PE outcomes using MiSeq data is a newer concept and one tool is available, PE-Analyzer, with more likely to follow (Hwang et al., 2021). Sanger sequencing represents a cheap alternative for analyzing the composition of editing outcomes in bulk populations. Tools include Synthego’s ICE (Inference of CRISPR Edits) (Hsiau et al., 2019) and Tracking Indels by Decomposition (TIDE) (Brinkman et al., 2014). Sanger Sequencing tools designed for CRISPR/Cas9 have been applied to PE experiments highlighting their versatility (Ferreira da Silva et al., 2022).

Given the challenges of editing the loci described in our work, a more complex system such as PE might be worth exploring in future studies. PE could be compared to CRISPR/Cas9 using the methods described to determine whether the trade-off between the relative simplicity of CRISPR/Cas9 is balanced by increased efficiency. Compared to the extensive characterization of CRISPR/Cas9, the optimization of PE is a growing field. A significant advance is the manipulation of mismatch repair pathways in PE (Chen et al., 2021; Ferreira da Silva et al., 2022). At present pegRNA design is the major determinant of PE efficiency (Anzalone et al., 2019). Finding the most efficiency combination of PBS and RT designs is an important preliminary step.

For those who will attempt HDR by CRISPR/Cas9 based on its relative simplicity and the robustness of the CRISPR/Cas9 recombinant components compared to PE, if an acceptable efficiency is attained after clonal isolation, the modifications we have validated may be employed to boost efficiency.

## 5 Conclusion

For SNVs for which a cut site can be generated at < 15 nucleotides, it may be simpler and faster to use CRISPR/Cas9 with protocol modifications to achieve HDR. For more challenging loci, such as those presented in this study, PE could be considered but would require more extensive optimization. For all genome editing systems the efficiency varies with cell model and target locus amongst other factors. Preliminary testing will inform the choice of system and protocol modifications. We propose a flowchart which could be used to guide the planning of CRISPR/Cas9 experiments to edit SNVs (Figure 4).

## Data Availability

The datasets presented in this study can be found online at https://www.ebi.ac.uk/ena with accession number PRJEB52736.
