# Supplementary material for: Optimizing CRISPR/Cas9 Editing of Repetitive Single Nucleotide Variants
Source: Front Genome Ed. 2022 Jul 5;4:932434. doi: 10.3389/fgeed.2022.932434 (PMC9294353; doi:10.3389/fgeed.2022.932434)
Supplement: Supplementary file 1 [file DataSheet1.docx]

Supplementary Material

**
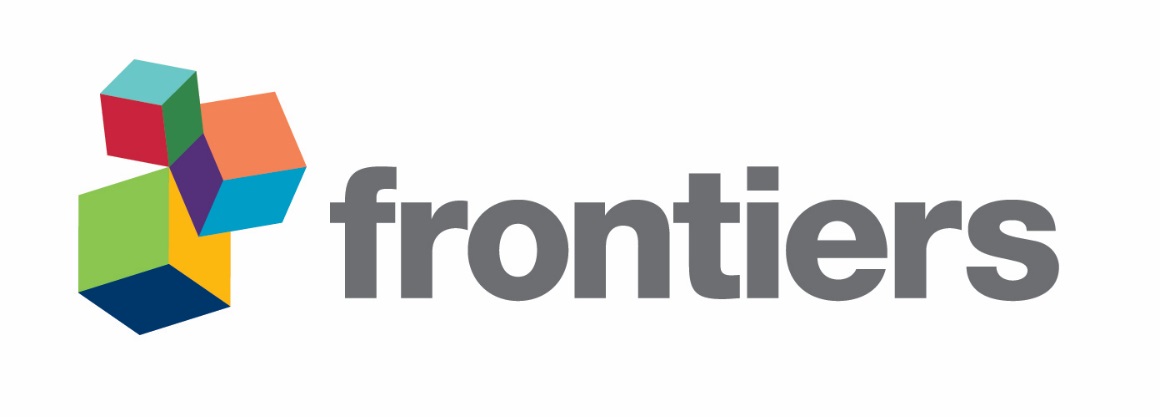
**

| **Modification** | **Mechanism(s) of action** |
| --- | --- |
| gRNA design (Song and Stieger, 2017) |  |
|  | Preferential binding of CRISPR/Cas9 |
| Asymmetric ssODN (Richardson *et al.*, 2016; Liang *et al.*, 2017; O’Brien *et al.*, 2019) | Influences annealing and release of strands being repaired |
|  |  |
| Blocking mutation in ssODN (Paquet *et al.*, 2016; Okamoto *et al.*, 2019) | Prevents cleavage of ssODN by Cas9 |
| Phosphorothioate modification of nucleotides (Papaioannou, Disterer and Owen, 2009; Gutierrez-Triana *et al.*, 2018) | Prevents degradation of ssODN |
|  |  |
| ssODN complementary to non-target strand (Richardson *et al.*, 2016) | Preferential binding of CRISPR/Cas9 |
| Cold shock (Guo *et al.*, 2018; Maurissen and Woltjen, 2020) | Not known, possibly accumulation of cells in G2/M phase or persistence of RNP |
| HDR enhancer (Pinder, Salsman and Dellaire, 2015; Maurissen and Woltjen, 2020), reviewed in (Bischoff *et al.*, 2020) | Inhibits NHEJ |
| Changing length of homology arms (Okamoto *et al.*, 2019) | Influences annealing and release of strands being repaired |
| DMSO (Stratigopoulos *et al.*, 2018) | Not known, possibly improved DNA access or cell cycle arrest |
| Cell cycle synchronization and RNP concentration (Lin *et al.*, 2014; Zhang *et al.*, 2017) | Favours cell cycle phases when HDR is active |

**Supplementary Table 1. Modifications to the CRISPR/Cas9 protocol to boost editing rates (reviewed in Liu, 2019).**

|  | **U-CH1 profiles** | | **Database profiles** | |
| --- | --- | --- | --- | --- |
| **Marker** | Allele 1 | Allele 2 | Allele 1 | Allele 2 |
| AMEL | X | Y | X | Y |
| CSF1PO | 10 | 11 | 10 | 11 |
| D13S317 | 11 | 13 | 11 | 13 |
| D16S539 | 12 | 13 | 12 | 13 |
| D18S51 | 15 | 15 | 15 | 15 |
| D21S11 | 28 | 29 | 28 | 29 |
| D3S1358 | 15 | 15 | 15 | 15 |
| D5S818 | 11 | 12 | 11 | 12 |
| D7S820 | 9 | 12 | 9 | 12 |
| D8S1179 | 10 | 15 | 10 | 15 |
| FGA | 20 | 21 | 20 | 21 |
| Penta D | 11 | 11 | 11 | 11 |
| Penta E | 7 | 10 | 7 | 10 |
| TH01 | 7 | 7 | 7 | 7 |
| TPOX | 8 | 11 | 8 | 11 |
| vWA | 17 | 17 | 17 | 17 |
| AMEL | X | X | X | X |

**Supplementary Table 2. STR (Short Tandem Repeat) analysis results for U-CH1, used in the study.**

| **Primer or Template** | **Sequence (5’ to 3’)** |
| --- | --- |
| *TP53* MiSeq forward |  |
|  | TCGTCGGCAGCGTCAGATGTGTATAAGAGACAGGGGTCAGAGGCAAGCAGAG |
| *TP53* MiSeq reverse | GTCTCGTGGGCTCGGAGATGTGTATAAGAGACAGTTGGGCCTGTGTTATCTCCT |
|  |  |
| *TP53* guide RNA | TGTTACACATGTAGTTGTAG |
| G245D_g3_ssODN1 | CTGTGTTATCTCCTAGGTTGGCTCTGACTGTACCACCATTCACTACAACTACATGTGTAACAGTTCCTGCATGGGCGACATGAACCGGAGGCCCATCCTCACCATCATCACAC |
| G245D_ g3_ssODN3 | CGCACTGGCCTCATCTTGGGCCTGTGTTATCTCCTAGGTTGGCTCTGACTGTACCACCATTCACTACAACTACATGTGTAACAGTTCCTGCATGGGCGACATGAACCGGAGGCCCATCCTCACCATCATCACAC |
| G245D_ g3_ssODN3 –no PAM | CGCACTGGCCTCATCTTGGGCCTGTGTTATCTCCTAGGTTGGCTCTGACTGTACCACCATCCACTACAACTACATGTGTAACAGTTCCTGCATGGGCGACATGAACCGGAGGCCCATCCTCACCATCATCACAC |
| G245D_ g3_ssODN3-RC | GTGTGATGATGGTGAGGATGGGCCTCCGGTTCATGTCGCCCATGCAGGAACTGTTACACATGTAGTTGTAGTGAATGGTGGTACAGTCAGAGCCAACCTAGGAGATAACACAGGCCCAAGATGAGGCCAGTGCG |
| R248Q_ g3_ssODN3-PT | C*G*CACTGGCCTCATCTTGGGCCTGTGTTATCTCCTAGGTTGGCTCTGACTGTACCACCATTCACTACAACTACATGTGTAACAGTTCCTGCATGGGCGGCATGAACCAGAGGCCCATCCTCACCATCATCAC*A*C |
| *TP53* Sanger sequencing forward | GCAGTAAGGAGATTCCCCGC |
| *TP53* Sanger sequencing reverse | TTGCCACAGGTCTCCCCAAG |
| *TBXT* MiSeq forward | TCGTCGGCAGCGTCAGATGTGTATAAGAGACAGTTCAGTGCCACCAATCCTGTA |
| *TBXT* MiSeq reverse | GTCTCGTGGGCTCGGAGATGTGTATAAGAGACAGATCCGCCTCTGTCCTTCTCA |
| *TBXT* ddPCR forward | GCCACCAATCCTGTATC |
| *TBXT* ddPCR reverse | TCAGGGAAGCAGTGG |
| *TBXT* ddPCR edited probe | [6FAM]TCATGCGCTGTGGACCC[BHQ1] |
| *TBXT* ddPCR unedited probe | [HEX]TCATGCGCTGTGGATCC[BHQ1] |
| *TBXT* Sanger sequencing forward | TTCAGTGCCACCAATCCTGTAT |
| *TBXT* Sanger sequencing reverse | CACTTGTATGGAGAATTCAAGG |
| *TBXT* guide RNA | GATCCCCAACTCTCACTATG |
| *TBXT* ssODN | TTCAGTGCCACCAATCCTGTATCTGTCTCCCTCAGATCATGCTGAACTCCTTGCATAAGTATGAGCCTCGAATCCACATAGTGAGAGTTGGGGATCCACAGCGCATGATCACCAGCCACTGCTTCCCTGAGACCCAGTTCATAGCGGTGACTGCTTATCAGAACGAGGAGGTGAGAAGGACAGAGGCGGAT |

* = Phosphorothioate bonds

**Supplementary Table 3. List of primers, guides, donors used in this study.**

***
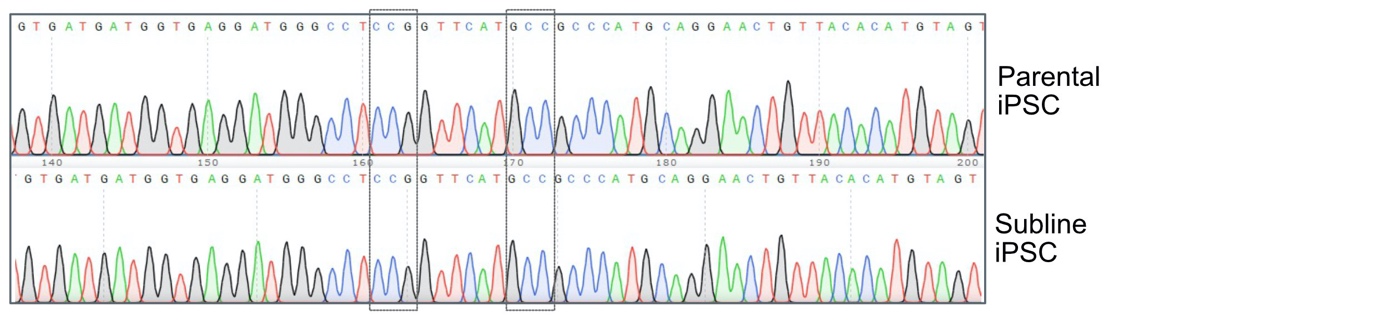
***

**Supplementary Figure 1. Sanger sequencing of TP53 from colony-picked iPSCs.** Representative Sanger sequencing traces of TP53 exon 7 for one of the five tested sublines: all sublines were free of SNVs in the TP53 sequence surrounding the G245D and R248Q loci (highlighted by boxes) and were wild type for the G245D and R248Q SNVs.


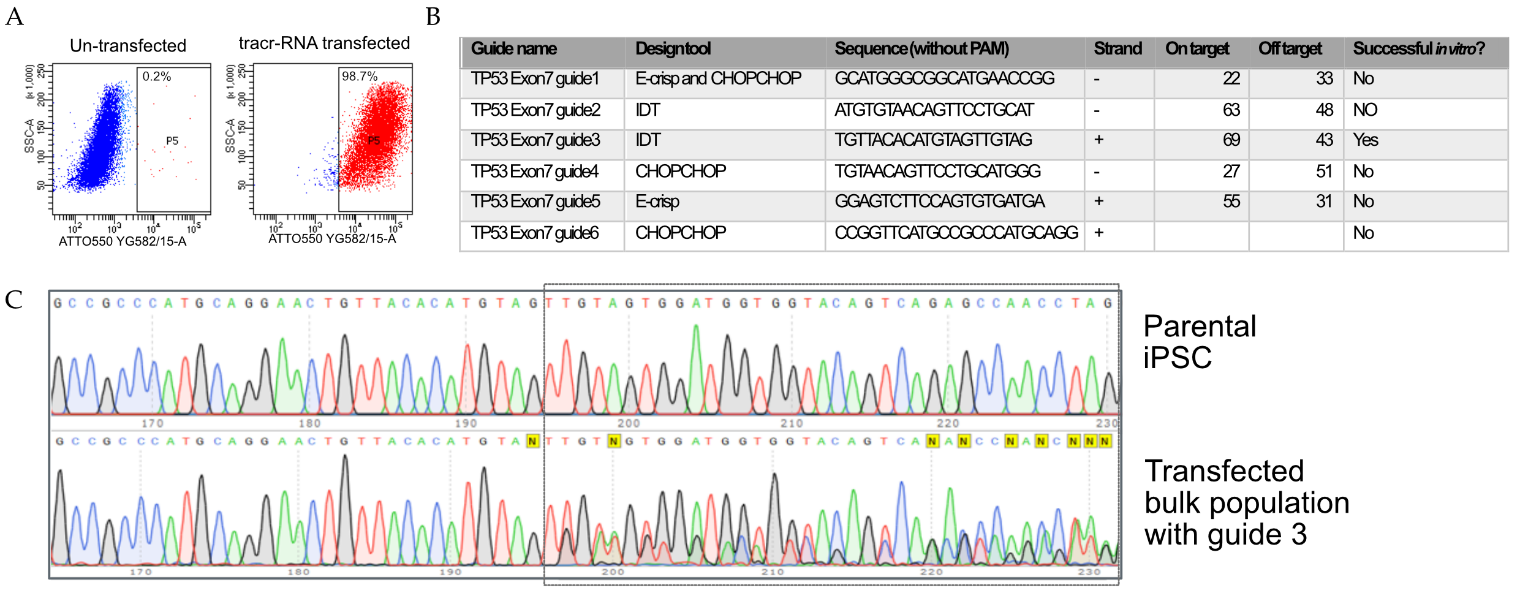


**Supplementary Figure 2. iPSC after transfection with RNP containing *TP53* exon 7 guide RNA 3.** (A) Dot plots showing 98.7% ATTO-550-positive cells by FACS after transfection with the gRNA-tracrRNA duplex. (B) Sequence and information on all designed and tested gRNAs. (C) Sanger sequencing traces of the parental iPSC showing the reference sequence (top) and the bulk population transfected with the successful gRNA (*TP53* exon 7 guide 3), showing evidence of repair by NHEJ at the predicted cut site (bottom).


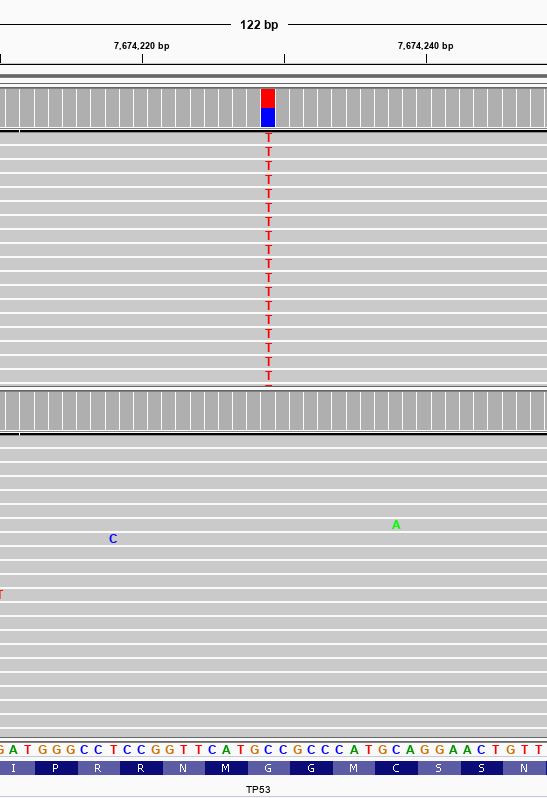


**Supplementary Figure 3. Integrated Genome Viewer capture of heterozygous knock-in of iPSC and wild type iPSC.**


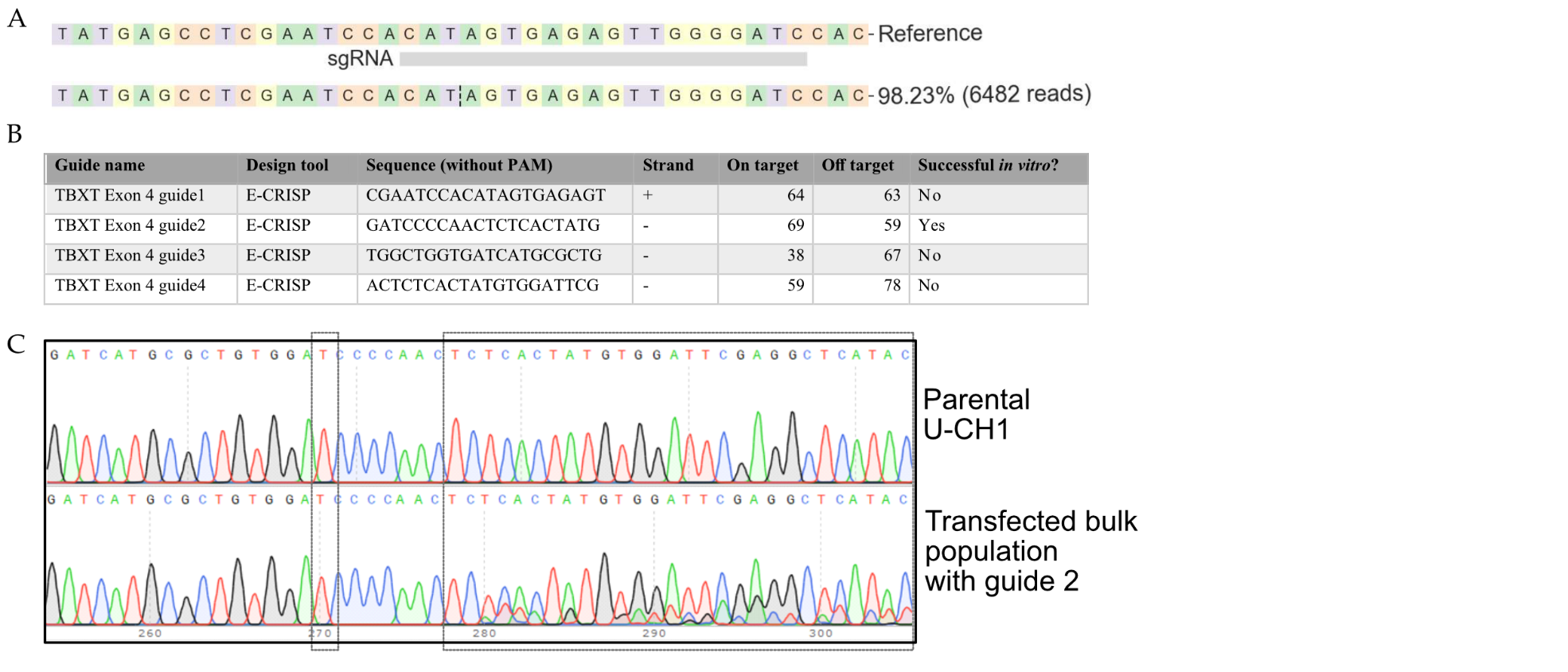


**Supplementary Figure 4. CRISPR/Cas9 gRNA design for U-CH1 chordoma cell line.** (A) MiSeq™ results from the U-CH1 parental cell line analysed by CRISPResso2 [38] showing a genetically pure starting population, homozygous for the variant allele at the G177D SNV. (B) Sequence and information on all designed and tested gRNAs. (C) Sanger sequencing traces of the parental U-CH1 cells showing the reference sequence (top) and the bulk population transfected with the successful gRNA, showing evidence of repair by NHEJ at the predicted cut site (bottom). Dashed box on the left highlights the G177D SNV, and on the right highlights the repaired region downstream of the cut site.


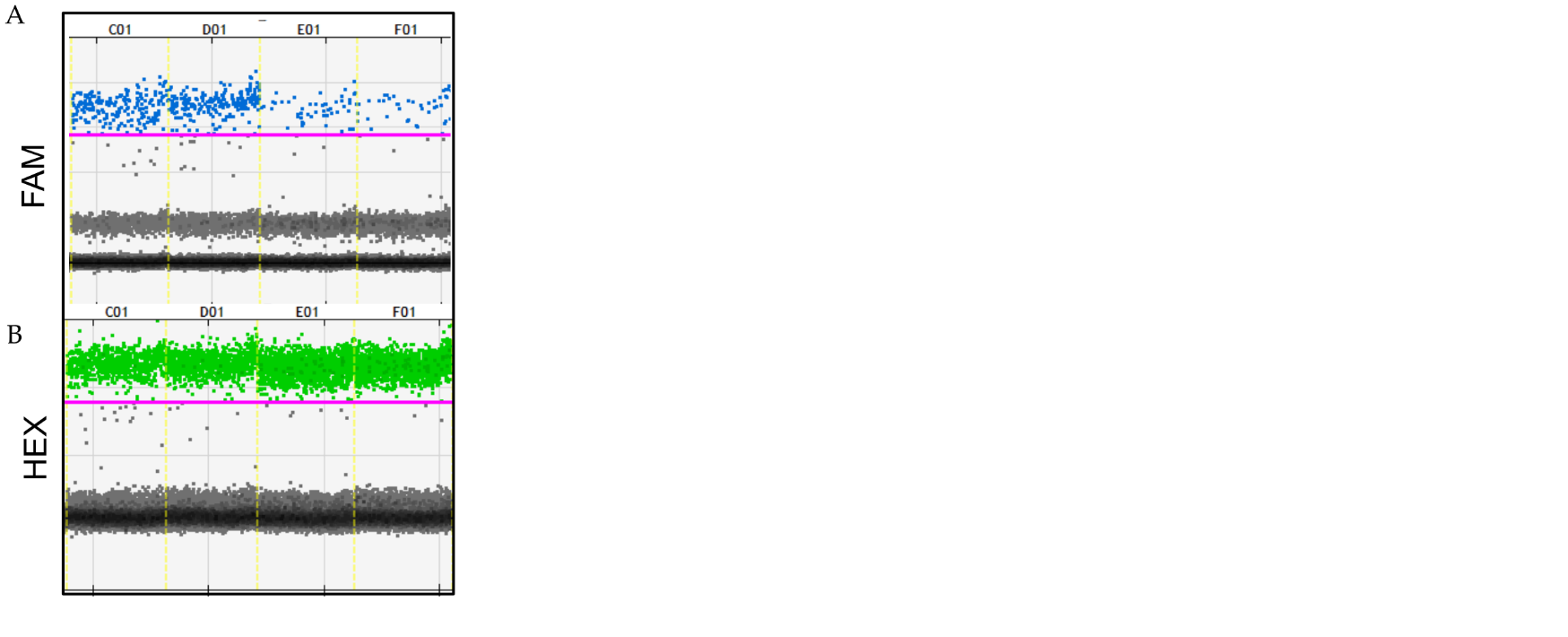


**Supplementary Figure 5. Dot plot of ddPCR assay for edited and unedited alleles in U-CH1.** (A) Blue droplets (FAM) represent the edited allele and (B) green droplets (HEX) represent the reference allele.

**
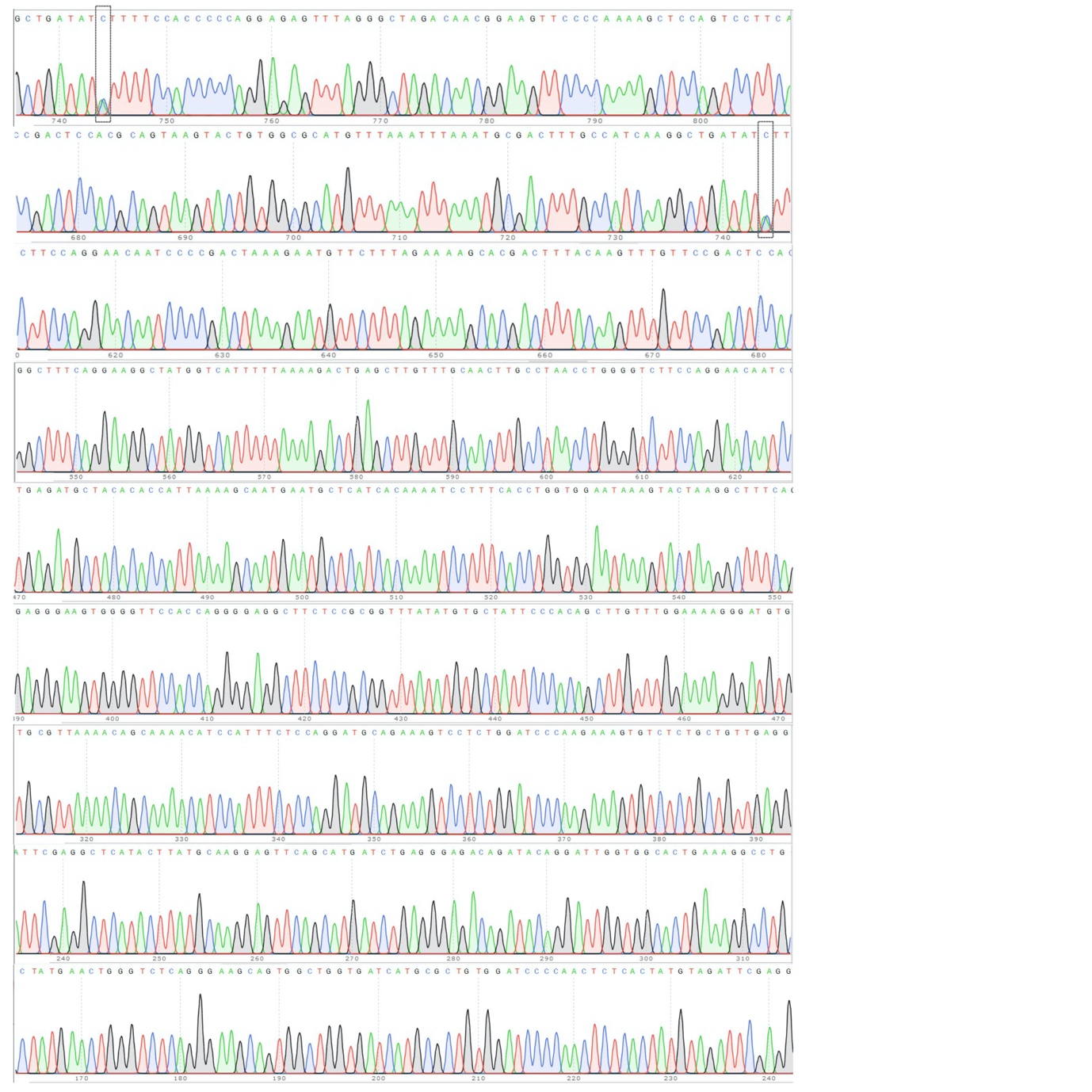
**

**Supplementary Figure 6. Quality assurance of edited U-CH1 clonal lines.** Sanger sequencing traces of 1,000 bp around the site of the edit in one heterozygous edited clone showing accurate editing of the G177D SNV in *TBXT* and no off-target alterations. Dashed boxes indicate the G177D SNV.
